# Hepatitis A Virus Genotype IB Outbreak among Internally Displaced Persons, Syria

**DOI:** 10.3201/eid2602.190652

**Published:** 2020-02

**Authors:** Malak Kaddoura, Rasmieh Allaham, Abdinasir Abubakar, Amani Ezzeddine, Amal Barakat, Peter Mala, Hassan Zaraket

**Affiliations:** American University of Beirut, Beirut, Lebanon (M. Kaddoura, A. Ezzeddine, H. Zaraket);; World Health Organization Country Office, Damascus, Syria (R. Allaham);; World Health Organization Regional Office for the Eastern Mediterranean, Cairo, Egypt (A. Abubakar, A. Barakat, P. Mala)

**Keywords:** hepatitis A virus, genotype IB, outbreak, Syria, internally displaced persons, viruses

## Abstract

In 2018, a hepatitis A virus outbreak was identified among internally displaced persons in Syria. Sequence analysis based on the viral protein 1/2A junction revealed that the causative virus belonged to genotype IB. A high displacement rate, deteriorated sanitary and health conditions, and poor water quality likely contributed to this outbreak.

Hepatitis A virus (HAV) is the leading cause of acute hepatitis infections worldwide, infecting ≈1.5 million persons annually (*1*). Symptoms, which are usually mild, include nausea, vomiting, abdominal pain, restlessness, body weakness, myalgia, loss of appetite, and fever. However, HAV may progress into fulminant liver failure, necessitating liver transplant. Generally, HAV is self-limiting (*2*). HAV (genus *Hepatovirus*, family *Picornaviridae*) is a nonenveloped virus with a single-stranded, positive-sense RNA linear genome (7.5 kb). The viral proten (VP) 1/2A junction (168 nt) is used to classify HAV into 6 genotypes: I–III (subgenotypes A and B) of human origin and IV–VI of simian origin (*3*). Genotype IA is the most commonly reported worldwide, whereas genotype IB is predominant in the Middle East (*4*–*6*).

On September 9, 2018, the governorate of Aleppo, Syria, informed the World Health Organization office in Syria that internally displaced persons (IDPs; displaced since early 2018) and local host community members in Tal Refaat, Fafin, and surrounding areas in the northwestern and western parts of Aleppo were experiencing a suspected hepatitis outbreak. The affected area included 17 locations in Azaz and Jabal Sem’an districts in western Aleppo (Appendix). Outbreak field investigation found sporadic cases of the disease among IDPs starting July 21, 2018; as of November 8, a total of 638 cases of suspected acute hepatitis infection had been reported. Most patients (98.59%) were <15 years of age and the rest 16–54 years of age. A total of 105 patients (16.5%) were admitted into the Fafin hospital; no fatalities were reported. No field investigations were performed in the first half of 2018 because of the crisis that led to weakness in the routine surveillance system.

A total of 48 unidentified serum and plasma samples were collected from 24 IDP children with suspected hepatitis and sent to the laboratory on October 29. The specimens originated from 3 locations in Syria: 13 from Fafin camp in Aleppo, 6 from eastern rural Daraa, and 5 samples from rural Quneitra. Even though the main outbreak was in the Aleppo governorate, Daraa and Quneitra were also experiencing a notable upsurge in reported cases of suspected acute hepatitis infection. For this reason, additional samples were collected from these governorates.

We analyzed the serum specimens by serology (total HAV antibodies and HAV IgM) using the enzyme-linked fluorescent assay VIDAS (bioMérieux Diagnostics, https://www.biomerieux-diagnostics.com) and the plasma specimens by real-time reverse transcription PCR (RT-PCR) for the detection of HAV (using the HAVNET protocol) and hepatitis E virus (HEV) (*7*). Seven samples had insufficient volume to perform both total HAV antibody and HAV IgM tests; thus, only the IgM test was performed. Overall, 19 plasma specimens were positive for HAV and none for HEV by PCR (Table). Eighteen serum specimens had detectable HAV IgM. All the specimens with sufficient volume (n = 17) were positive for total HAV antibodies. Of these, 5 were from past infections, as indicated by the negative HAV PCR and HAV IgM results. One patient had detectable HAV vRNA but negative HAV IgM, which indicates the early start of acute infection (*8*). This patient’s serum was positive for total HAV antibodies, indicating a previous exposure with a current breakthrough infection.

**Table:** Serology and PCR analysis of serum and plasma specimens from the suspected hepatitis outbreak*

Lab ID	District	Serology			Molecular analysis		
		HAV IgM	HAV total		HAV vRNA	HEV vRNA	Genotype
1	Fafin	+	+		+	–	ND
2	Fafin	+	+		+	–	IB
3	Fafin	+	+		+	–	IB
4	Fafin	+	+		+	–	ND
5	Fafin	+	+		+	–	ND
6	Fafin	+	+		+	–	IB
7	Fafin	+	+		+	–	IB
8	Fafin	+	+		+	–	ND
9	Fafin	–	+		–	–	–
10	Fafin	+	+		+	–	ND
11	Fafin	+	NS		+	–	ND
12	Fafin	–	+		–	–	–
13	Fafin	–	+		–	–	–
14	Quneitra	+	+		+	–	ND
15	Quneitra	–	+		+	–	ND
16	Quneitra	–	+		–	–	–
17	Quneitra	+	+		+	–	ND
18	Quneitra	+	+		+	–	IB
19	Daraa	+	NS		+	–	ND
20	Daraa	+	NS		+	–	ND
21	Daraa	+	NS		+	–	IB
22	Daraa	+	NS		+	–	ND
23	Daraa	+	NS		+	–	ND
24	Daraa	–	NS		–	–	–

*HAV, hepatitis A virus; HEV, hepatitis E virus; ND, not determined; NS, no sufficient volume; +, positive; –, negative.

We successfully sequenced the VP1/2A region for 6 specimens (Table) and used ClustalW in BioEdit 7.0 to align the sequences (*9*). Sequence-based genotypes were inferred by comparing the obtained sequences with genotype reference and contemporary strains obtained from GenBank. The phylogenetic analysis indicated that all Syria specimens belonged to genotype IB.

The Office for the Coordination of Humanitarian Affairs reported that, as of September 3, 2018, a total of 23,279 families (107,083 persons) were displaced from Afrin to Tel Refaat, Fafin, and surrounding villages. These IDPs were in addition to 4,766 families (38,843 persons) in the host community. Tents, destroyed or empty dwellings, schools, mosques, and warehouses were used as collective shelters but are relatively distant from active frontlines. IDPs had restricted freedom of movement and no access to proper sanitation facilities as a result of infrastructure damage; 70% of the population rely on water trucking services, and 30% live on less than 20 L of water per day. Moreover, 88% of the respondents reported accumulation of solid waste in their areas (World Health Association, unpub. data). A health assessment in the Afrin district found that 153 of 180 (85%) assessed communities have no access to health services. Vaccination campaigns have been largely suspended since November 2016. These poor living and health conditions render the IDPs highly prone to vaccine-preventable diseases, including hepatitis A, measles, polio, and cholera (*10*). Although water testing for HAV was not possible in the affected areas, deterioration in water quality was reported down the supply chain and may have contributed to this outbreak (World Health Association, unpub. data).

In summary, we report a large outbreak of hepatitis among IDPs in Syria. Laboratory testing confirmed current HAV IB infection among most screened patients. The high displacement rate, deteriorated sanitary and health conditions, and poor water quality may have all contributed to the increased HAV reports among this population.

Appendix Locations of hepatitis A virus genotype IB outbreak among internally displaced persons, Syria.
