# Supplementary material for: Hepatitis A Virus Genotype IB Outbreak among Internally Displaced Persons, Syria
Source: Emerg Infect Dis. 2020 Feb;26(2):369–71. doi: 10.3201/eid2602.190652 (PMC6986849; doi:10.3201/eid2602.190652)
Supplement: Appendix — Locations of hepatitis A virus genotype IB outbreak among internally displaced persons, Syria. [file 19-0652-Techapp-s1.pdf]

Article DOI: https://doi.org/10.3201/eid2602.190652

Hepatitis A Virus Genotype IB Outbreak among Internally Displaced Persons, Syria

Appendix

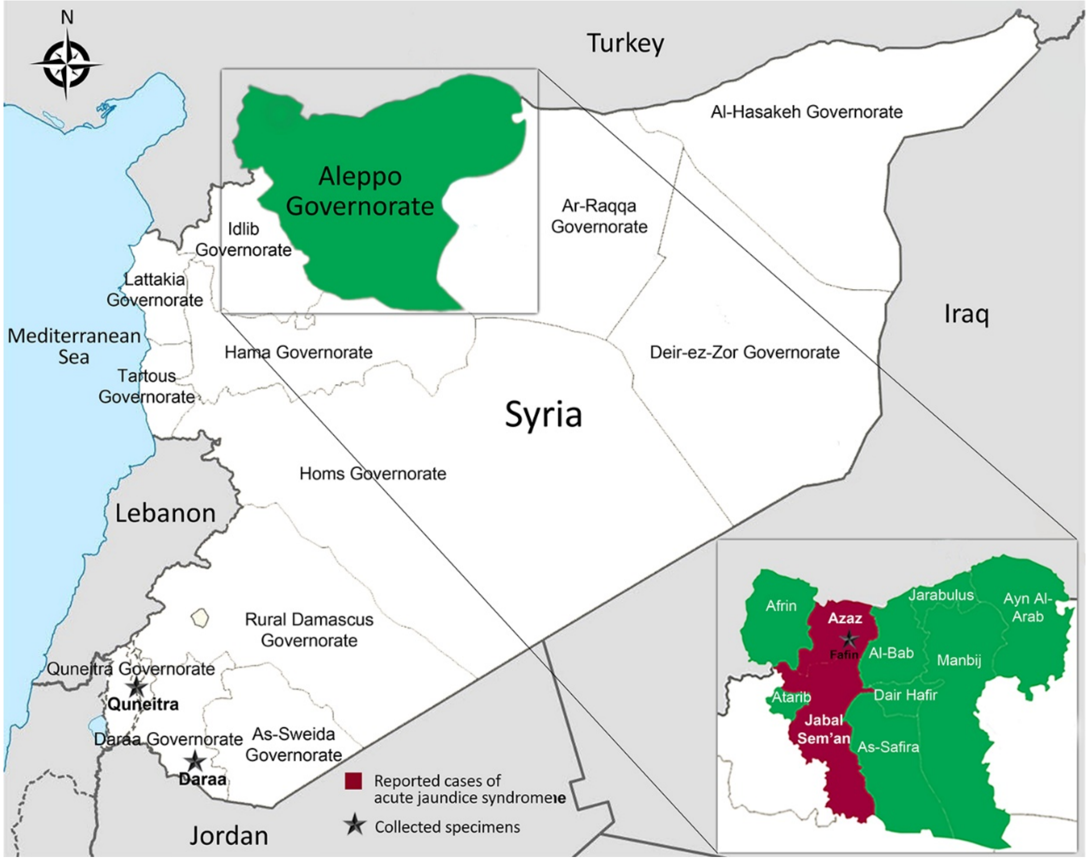

Appendix Figure. Map of Syria showing the locations of the hepatitis A outbreak.
